# Neuropathology, Localized Proteomics, and Imaging of the Choroid Plexus across the Alzheimer's Disease Continuum

**DOI:** 10.1002/alz70855_105614

**Published:** 2025-12-24

**Authors:** Dominique Leitner, Chenyang Li, Huize Pang, Mary Bruno, Arline Faustin, Orrin Devinsky, Evgeny Kanshin, Beatrix Ueberheide, Youssef Zaim‐Wadghiri, Jiangyang Zhang, Yulin Ge, Thomas Wisniewski

**Affiliations:** ^1^ NYU Grossman School of Medicine, New York, NY, USA; ^2^ New York University Grossman School of Medicine, New York, NY, USA

## Abstract

**Background:**

The choroid plexus (ChP), a highly vascularized structure within the ventricles, plays a vital role in cerebrospinal fluid (CSF) production and metabolic waste clearance, essential for brain homeostasis and function. *In vivo* imaging studies have identified age‐ and Alzheimer's disease (AD)‐related ChP changes, including increased size, vascular degeneration, and cyst‐like formations. We previously identified proteomic differences in severe AD cases compared to controls, associated with a shift in cell energy metabolism. However, investigation of underlying mechanisms remains limited. We sought to characterize ChP alterations in AD using histopathology and proteomics, focusing on blood‐CSF barrier integrity, vascular membrane changes, Aβ and tau accumulation, and epithelial and stromal tissue alterations.

**Method:**

We evaluated the ChP by MRI, neuropathology, and proteomic analyses in AD and control cases. MRI findings were compared to our prior study on ChP using the HCP‐aging dataset (PMID: 39639335). Neuropathology characterization included Aβ, tau, endothelial marker CD31, epithelial marker AQP1, basement marker collagen IV (COL4). To follow up on our previous proteomic studies and identify protein changes across the AD continuum, we evaluated microdissected ChP from autopsy paraffin embedded tissue adjacent to the hippocampus at the level of the lateral geniculate nucleus, followed by label‐free quantitative mass spectrometry. Protein differences and complementary histology characterization and quantification of top proteins were evaluated, with correlations to case history.

**Result:**

Proteomics identified protein differences across the AD continuum, with some proteins previously described as well as novel proteins. Functional associations with altered proteins were evaluated by gene ontology (GO) enrichment. After comparative analyses with other brain tissue and biofluid proteomic datasets, top protein candidates were identified and characterized by histology.

**Conclusion:**

We performed an initial characterization of the ChP by MRI and neuropathology, and identified proteomic differences in AD. Our results provide valuable insights into molecular mechanisms, novel diagnostic biomarkers, and potential therapeutic strategies.

